# Hazard Assessment of Urban Trees along the Bagmati River Corridor: A Case Study from Kathmandu Metropolitan City, Ward Number 8

**DOI:** 10.1155/2023/6632577

**Published:** 2023-10-26

**Authors:** Jun Shapkota, Jeetendra Gautam

**Affiliations:** Agriculture and Forestry University, Bharatpur, Nepal

## Abstract

Urban trees are extremely significant and provide numerous advantages for both the environment and people. In order to provide a healthy and sustainable environment, green spaces have long been seen as a crucial component of contemporary cities. Nevertheless, as urbanization and population growth have accelerated, environmental challenges have become a major worldwide issue. This study mainly focuses on the assessment of hazardous trees along the Bagmati river corridor and documents major tree species for urban forestry. The study was conducted in ward number 08 of Kathmandu metropolitan city. Total enumeration was carried out by recording information on trees through direct observation of the whole site. The International Society of Arboriculture (ISA) tree hazard evaluation method was used as a tree risk assessment tool. A total of 74 trees were recorded from the study site. *Grevillea robusta*, *Cinnamomum camphora*, *Ficus bengalensis*, *F. religiosa*, and so on were the major species planted along the Bagmati corridor. The trees were categorized in varying degrees of hazard. Forty-seven trees were less hazardous, 24 were semihazardous, and 3 were hazardous with hazard rates of 2.91, 6.04, and 9, respectively. *Jasminum nudiflorum* was the only hazardous species recorded followed by semihazardous species such as *Morus alba* and *F. religiosa* among other species, and *Eucalyptus camaldulensis*, *Citrus limon*, *Psidium guajava*, *Alnus nepalensis*, and so on were least hazardous tree species. The hazardous tree in urban areas increases the risk to people and public as well as private properties. There is a need for the removal of such hazardous trees and planting the right species at the right time. Moreover, regular inspection and a clear policy for urban trees are needed.

## 1. Introduction

The term “urban forestry” was first used in 1965 by [1] as part of a study on the successes and failures of a municipal tree planting program. Urban forestry is a concept that originated in North America in response to the growing importance of tree-dominated areas as well as human pressure on green spaces [2]. Urban forestry is the practice of forestry that is defined as the management of publicly and privately held lands in and near metropolitan centers. It includes parks, gardens, roadside plantation, stream bank plantation, and plantation in private property and forest near urban areas [3]. Urban forests contribute in sustainable development of cities through improving quality of life and environmental qualities [4]. In this type of forestry, trees are grown primarily for ornamental and flowering trees and shrubs, not for the production of fuel, food, or timber, to serve as recreation forests for urban inhabitants [5].

Urban parks are seen as significant drivers of the sustainable development of cities in both developed and developing nations. They provide a wide range of advantages to locals and visitors, including recreational activities, fresh air, aesthetically appealing features, and ecological purposes [6]. Urban green and blue areas that are easily accessible and of the highest quality enhance the local population's health significantly (European Environment Agency [7]). Green spaces increase biodiversity, reduce noise levels, and improve air quality [8, 9].

Urban and periurban forestry (UPF) has become a significant concern for policymakers and planners because of increasing urbanization [3]. Urbanization, which is the term for the movement of people from rural to urban regions, began in the Kathmandu valley in the late 1950s, but since 1970, the increase has been unmanaged [10]. In Nepal, the practice of urban forestry dates back to the Malla reign by King Jayasthiti Malla (1380–1395 AD) issuing an order to his officials and commoners to plant trees alongside walking streets and wells [11].

A typical 22% and 17%, respectively, of the total municipal area in Hill and Terai are covered by forests. In a mountainous area, it encompasses 30% of the entire municipal territory. The coverage in the Kathmandu valley is 3% [12]. In Kathmandu, the Department of Road (DOR) has been appointed to plant trees along the sides of the roads, while DOF is the organization with the necessary expertise in tree management and silviculture [13]. Integrative, strategic, multipurpose, transdisciplinary, and social inclusivity are among the concepts of urban forestry [14]. In recent years, the government has emphasized urban forestry through its various programs, including “Nepal Clean Environment Grand Expedition 2075 AD” and the Forest Decade Program (2014–2023) [3].

Despite the importance, environmental assets in general are frequently the ones that are most neglected. This is because the majority of poor nations often lack a scientific grasp of how urban trees, parks, and gardens benefit people [6]. Also, as urban areas increase, importance of benefits of urban forest as well as challenges to their conservation and maintenance will increase [15]. Urban trees are more likely to be site-stressed due to a number of factors. Most urban trees survive on construction-altered soils that may be compacted, poorly drained, high in clay, sand, or gravel, very alkaline, or littered with construction debris [16].

Despite various urban forestry programme conducted by DoF in Nepal, they still possess threats to people and properties commonly known as hazard. Hazards are things, conditions, or situations that have the potential to cause harm [17]. Tree hazards could be because of defects in trees, dead or dying trees, dead branches from living trees, or living trees that are unstable owing to structural flaws or other causes that are close to people or other property (a target) and might result in property damage, human harm, or even death in the case of failure [18, 19]. A tree defect is defined as a fault, flaw, or abnormality of the normal tree structure and function resulting in inadequate performance or failure [20]. Defects could be seen in the crown, branch, trunk, and roots.

Due to the usage of land for business and habitation uses, Kathmandu, the capital and most populated city of Nepal, lacks green parks and trees. As of 2017, just 3% of Kathmandu was covered with forest, according to the National Urban Development Strategy released by MoUD [12]. In addition, the shift of multiple land uses within the city as well as the neighboring urban fringes, together with industrialization in these regions, caused the decline of existing tree cover [11]. Thus, this study has been conducted to identify the planted tree species along the Bagmati river corridor and the defects in the tree species and hazards caused by them. This study also helps environmental managers and planners to understand and take into account the less tangible values that people derive from contact with nature.

## 2. Materials and Methods

### 2.1. Study Site

This study is carried out in ward number 08 of Kathmandu metropolitan city (KMC), along the Bagmati river corridor of the Kathmandu district. Generally, the Kathmandu district lies from 27°27′N to 27°49′N latitude and 85°10′E to 85°32′E longitude with an area of 433.61 km^2^ (167.42 sq. mi).

The area covered by ward number 08 is 2.538 km^2^, and the Bagmati river in the ward lies from 27°42′44″N to 27°42′20″N latitude and 85°21′34″E to 85°20′57″E longitude with an elevation of 2,740 m (8,990 ft) covering a length of 2.34 km. The annual temperature of the district varies from 19°C to 30°C during autumn and from 2°C to 17°C during winter, whereas the climate varies from subtropical to temperate. The annual precipitation is about 1,400 mm which falls mostly from May to September, with 80% during monsoon [21, 22].

**Figure 1:**
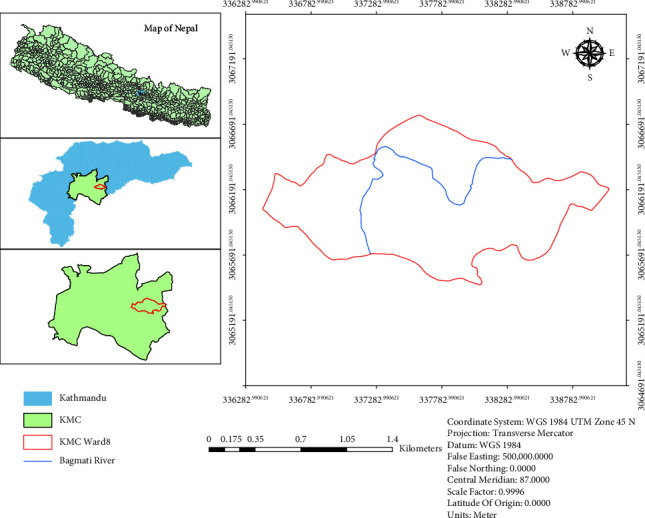
Map of Nepal showing Bagmati river inside KMC ward number 8.

The map of the study area is shown in Figure 1.

### 2.2. Sampling Method

Total enumeration of trees of the whole study site was done through direct observation. Information about trees (name of species, coordinates, girth of the tree, height of the tree, and defects) was recorded through direct observation of the site. The girth was measured using a diameter tape (cm) at diameter at breast height and height using a rangefinder (m).

### 2.3. Hazard Assessment

Guidelines from the book “A Photographic Guide to the Evaluation of Hazard Trees in Urban Areas” [23] was used to identify the hazardous tree. The steps given in the book are as follows:

- Identification of trees
- Formulation of evaluation parameters
- Identification of the tree's structural defects and affected structural components likely to fail
- Summarization of defects and the rank likelihood of structural failure
- Identification of the target in danger
- Summarization of hazard rating

Taking the average index, the sample site was classified as hazardous, semihazardous, and less hazardous.

Ms-Excel and ArcGIS were used to analyze data. Data were presented in the form of tables, charts, and diagrams. The International Society of Arboriculture (ISA) tree hazard evaluation method [23] was used. Hazard rating which is a summation of three components is shown as follows:

(1) $$\begin{array}{c} \text{failure potential}+\text{size of defective part}+\text{target rating}=\text{hazard rating}. \end{array}$$

Each component has a four-point rating system, for a combined total of twelve points, which is the maximum hazard rating. Components are as follows:

(A) *Failure Potential*. The potential of a tree or tree part to fall before another inspection is called failure potential. On basis of the presence of a type of defect, trees are classified as low failure potential to high failure potential and the value is assigned from 1 to 4, respectively:
  1. *Low*. This includes live trees without visible defects
  2. *Medium*. This includes live trees with only minor defects such as wounds that do not impair tree structure and trees with slight decay
  3. *High*. This includes live trees with moderate defects and decayed trees where sound wood shell thickness is at or near the minimum preferred amount
  4. *Severe*. This includes dead trees, live trees with major defects, and decayed or burned trees with less than acceptable remaining sound wood
(B) *Size of Defective Part.* Value is assigned from 1 to 4 depending upon the following size categories of defective parts:
  1. Less than 6 inches (<15 cm in diameter)
  2. 6 inches–18 inches (15–45 cm in diameter)
  3. 18 inches–30 inches (45–75 cm in diameter)
  4. Greater than 30 inches (>75 cm in diameter)
(C) *Target Rating.* This rating is carried out on the basis of target potential which is the potential of a tree or its part to hit a person or valuable property. The score is assigned on basis of the use of an area that a tree or its parts could normally reach if they fail.
  1. *Occasional Use.* This includes trees present around features with limited use and short use lengths such as garbage cans, campground signs, or fences
  2. *Intermittent Use*. This includes trees present around features with moderate use and short use lengths such as water sources or waste disposal stations
  3. *Frequent Use.* Trees present around developed tent sites, toilets, parking spurs, and other high-occupancy sites are under this category
  4. *Constant Use.* Trees present around the main roads are under this category

Table 1 shows the hazard rating scores for categorization of trees and sites.

## 3. Results and Discussion

### 3.1. Major Tree Species Listed in the Study Area

In the study site, we recorded 74 total trees of 22 different species that occurred in the sample site. *Grevillea robusta* was seen maximum followed by *Cinnamomum camphora*, whereas only one number of species such as *Phyllanthus emblica*, *Abies procer*a, and *Morus alba* were recorded.  Table 2 shows the species types recorded throughout the site. Also, saplings of *Prunus cerasoides*, *Ficus elastica*, *Mangifera indica*, *Eucalyptus camaldulensis*, *Ficus bengalensis*, and *Cinnamomum camphora* planted along the corridor were seen.

The results align with the findings of a study conducted by in Pakistan and Gautam et al. [24] in Kathmandu, which reported that *Grevillea robusta* was the most commonly planted tree species in urban areas, followed by *Cinnamomum camphora*, *Ficus* spp., *Eucalyptus camaldulensis*, and so on. The major purpose of this urban plantation seems for ornamentation as well as to maintain the urban ecosystem intact. Besides, road site trees ultimately fulfilled the amenity, shelter, wind firm, and evergreen and urban park [25]. This finding is also supported by a study by Knaus et al. [26] which found that certain tree species such as *Calliandra hematocephala* and *Juniperus chinensis* are more commonly planted in urban areas due to their aesthetic value.

### 3.2. Girth Distribution of Species

**Figure 2:**
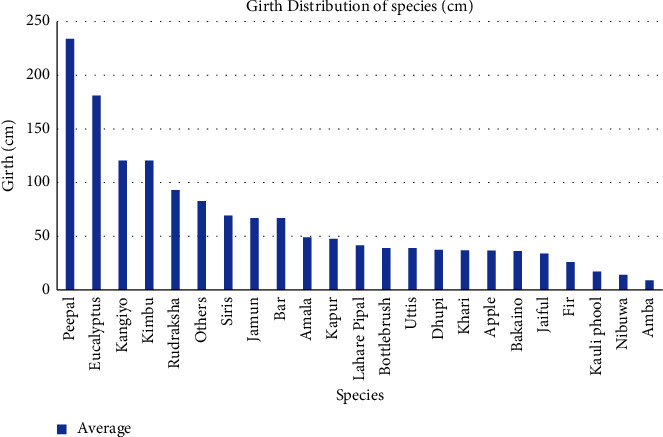
Average girth distribution of species in the study site.

In the study site, it was found that the highest girth was of Peepal (*Ficus religiosa*), i.e., 233.8 cm followed by *Eucalyptus camaldulensis*, i. e., 181 cm, both Kangiyo (*Grevillea robusta*) and Kimbu (*Morus alba*) with similar girth, i.e., 120.5 cm and so on, while the lowest girth was of Amba (*Psidium guajava*), i.e., 9cm followed by Nibuwa (*Citrus limon*) of 14 cm and Kauli phool (*Pileostegia viburnoides*) of 17 cm and so on, as shown in Figure 2.

### 3.3. Height Distribution of Species

**Figure 3:**
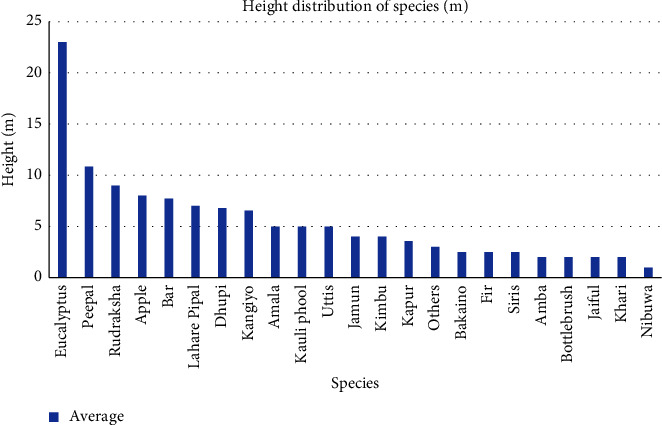
Average height distribution of species in map.

In the study site, *Eucalyptus camaldulensis* had an average height of 23 m, recorded as the highest, followed by *Ficus religiosa* 10.83 m and *Elaeocarpus sphaericus* 9 m and the lowest was of Nibuwa (*Citrus limon*), i.e., 1 m followed by Khari (*Celtis australis*), Jaiful (*Jasminum nudiflorum*), Bottlebrush (*Calliandra hematocephala*), and Amba (*Psidium guajava*), all four species having average height of 2 m as shown in Figure 3.

### 3.4. Tree Hazard Rating with respect to the Species

A hazard rating was given for each tree of each species, and average hazard rating was calculated for each species. Using this hazard rating of trees, species were classified as less hazardous, semihazardous, and hazardous trees.

**Figure 4:**
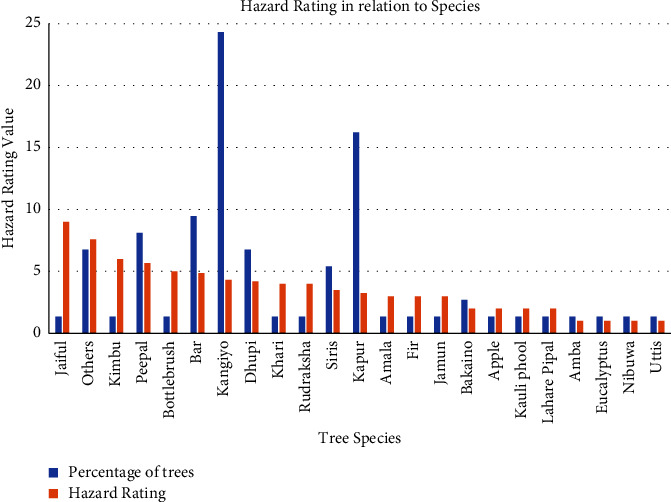
Hazard rating with respect to tree species.

Out of the total 23 tree species recorded, Jaiful (*Jasminum nudiflorum*) was the only hazardous species with 9 as hazard rating, whereas Amba (*Psidium guajava*), *Eucalyptus camaldulensis*, Nibuwa (*Citrus Limon*), and Uttis (*Alnus nepalensis*) had less hazard rating (1) which can be seen in Figure 4. The average hazard rating for less hazardous, semihazardous, and hazardous species was obtained as 2.38, 5.38, and 9, respectively.

### 3.5. Tree Hazard Rating with respect to the Hazardous Tree

A hazard rating was given for each tree. Using this hazard rating of trees, trees were classified as less hazardous, hazardous, and semihazardous.

**Figure 5:**
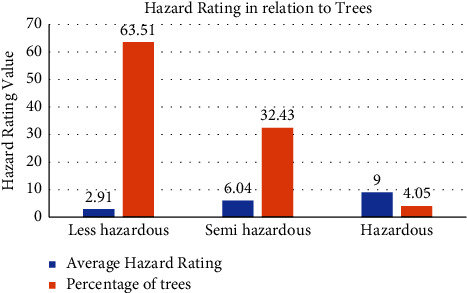
Tree hazard classification based on hazard rating.

Out of the total 74 trees recorded, 47 (63.51%) trees were less hazardous, 24 (32.43%) were semihazardous, and 3 (4.05%) trees were hazardous. The average hazard rating for less hazardous, semihazardous, and hazardous trees was obtained as 2.91, 6.04, and 9, respectively, which can be seen in Figure 5.

Our study showed a variety of possible tree hazards along the Bagmati river corridor, including structural defects, root damage, and pests and diseases. The majority of the trees surveyed had some degree of decay or rot, increasing the likelihood of failure during severe wind or storm occurrences. Furthermore, several trees were shown to have weakened root systems as a result of soil compaction or erosion, which might increase their risk of failure.

Results shown by [24] identified a range of tree species in the area, many of which were hazardous and needed to be removed immediately for public safety.

Moreover, our findings are consistent with several studies that have identified defects (knots, splits, hollowness, and so on) as common tree hazards in urban areas. A study by Ding et al. [27] in some major parks of Hongkong found that nearly half of the urban trees had shown some degree of defect While another study by Liu et al. [28] found that stem and root damage was the most common cause of tree failure in an urban park in Hong Kong.

**Figure 6:**
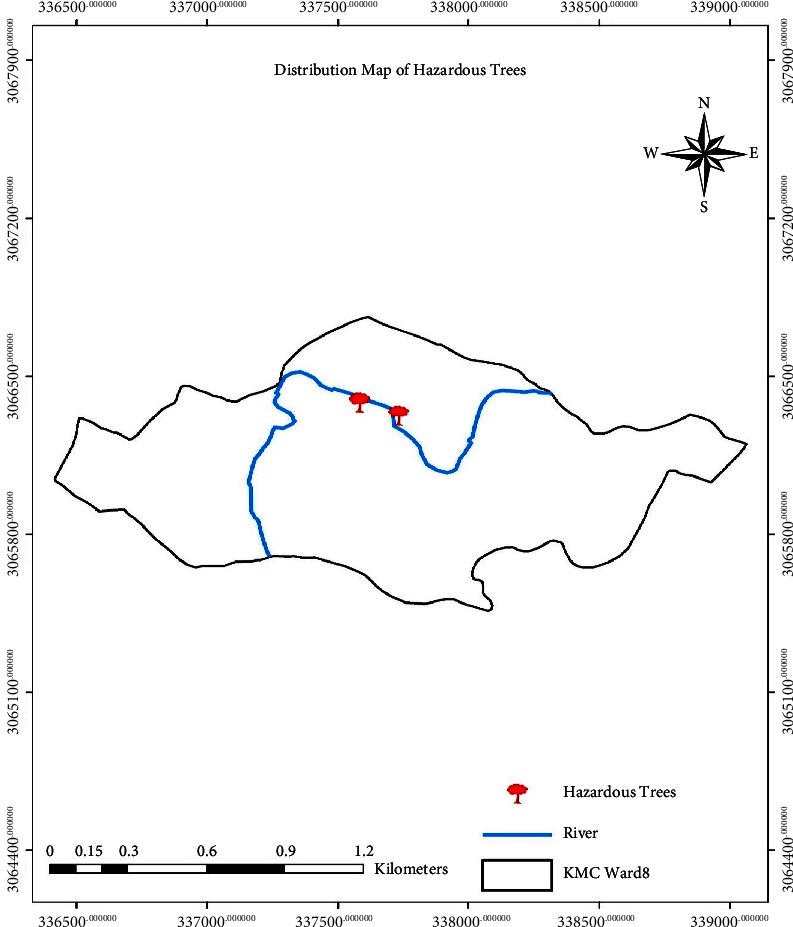
Distribution map showing hazardous trees.

**Figure 7:**
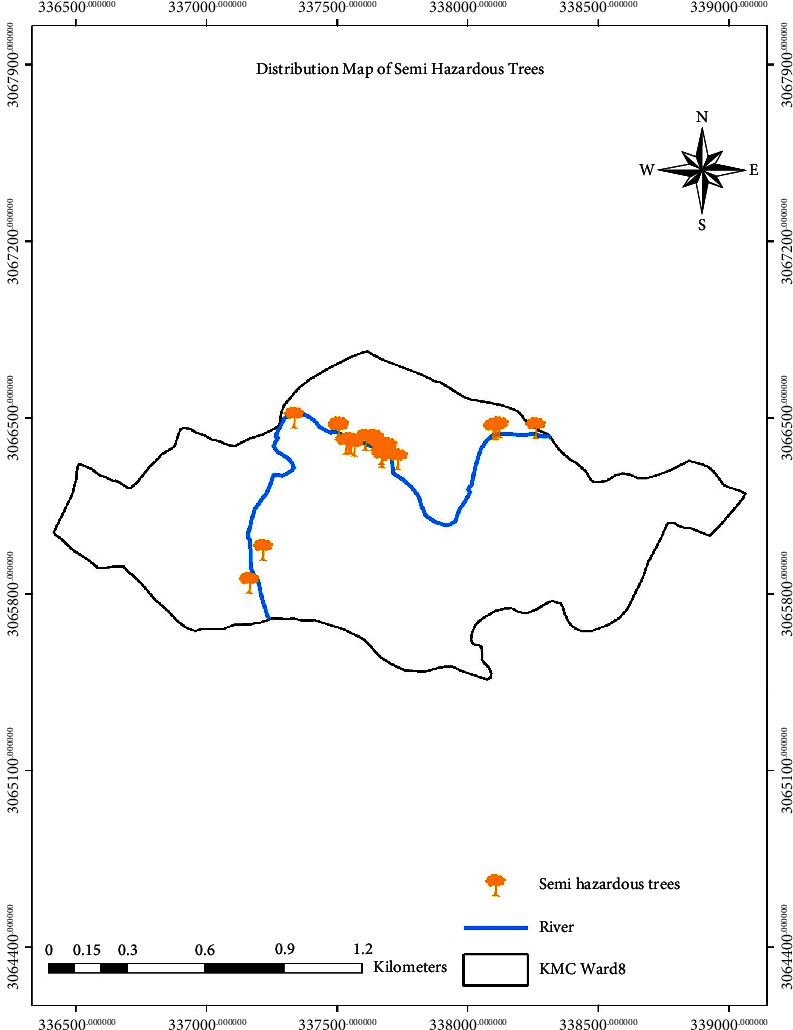
Distribution map showing semihazardous trees.

**Figure 8:**
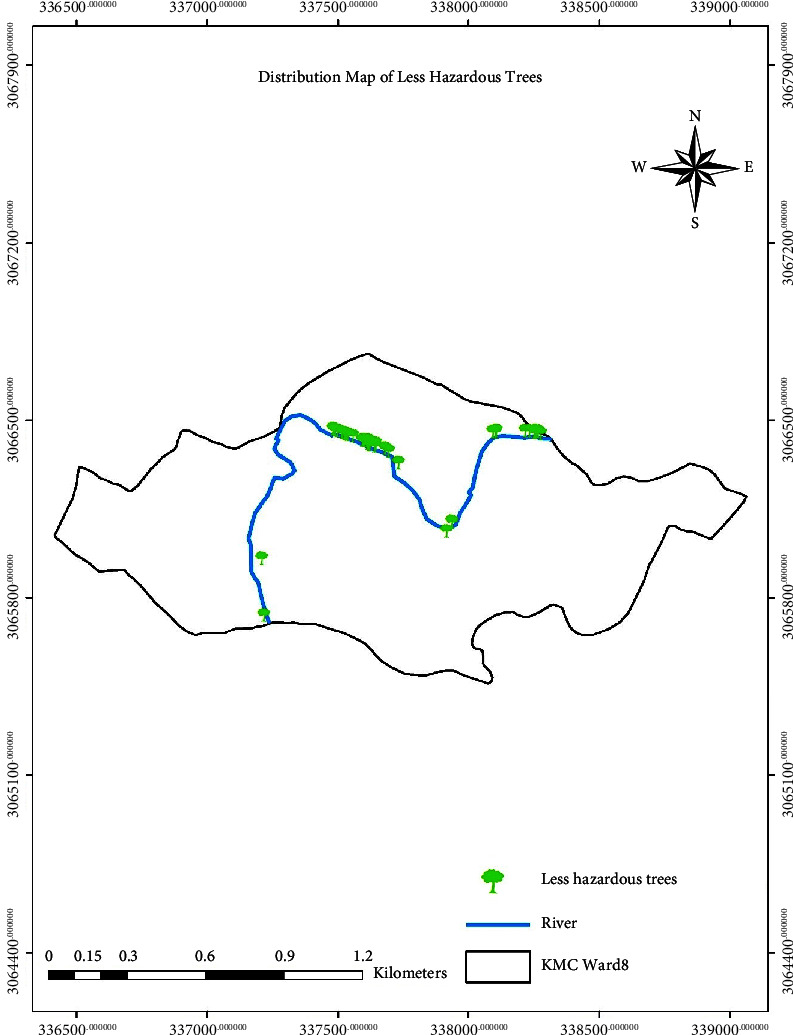
Distribution map showing less hazardous trees.

The distribution of trees based on hazard rating is shown in Figures 6–8, which shows the distribution of hazardous, semihazardous, and less hazardous trees, respectively.

## 4. Discussion

Our study found a significant number of trees with structural defects and symptoms of decay, posing a serious risk to public safety and property. The finding is consistent with the findings of many urban studies. For example, Leers et al. [29] conducted a comprehensive analysis of urban trees in Melbourne, Australia, and examined six indicators of urban tree establishment, emphasizing the importance of frequent tree inspections and maintenance for addressing structural defects.

The weakened root systems of many trees along the Bagmati river corridor, which are frequently caused by soil compaction and erosion, resonate with concerns raised in studies such as Wattenhofer and Johnson's [30] study on the effects of young urban tree death on future success and urban development on tree root health. They discovered that soil compaction can limit root growth and water infiltration, making trees more susceptible to windthrow and uprooting.

Our risk assessment approach, which takes into account both the likelihood as well as consequences of tree failure, is consistent with best practices in urban tree management [31]. Prioritizing trees near high-traffic areas, buildings, and infrastructure for management is consistent with recommendations of Pokorny et al. [16], in their study on urban tree risk management. This particular strategy can more efficiently allocate resources to mitigating the most significant risks and hazards. It is important for the Kathmandu district's relevant authorities to monitor and address these issues in order to sustain healthy trees in urban areas.

## 5. Conclusion

A total of 74 trees of 23 different species were recorded along the Bagmati river corridor of KMC ward number 8. Major plant species along the Bagmati river corridor were Indian Gooseberry, Chinaberry tree, Banyan, Red Powderpuff, Juniper, Noble Fir, Winter Jasmine, Black Plum, Silk oak, Camphor tree, Honeyberry, Mulberry, Cottonwood Poplar, Sacred Fig, Stone fruit, and so on. Saplings of plants such as *Eucalyptus*, *Prunus*, and *Mangifera* were also seen along the river corridor. *Jasminum* was the only hazardous tree followed by other semihazardous species *Morus*, *F. religiosa*, and so on, among other tree species, and *Eucalyptus*, *Citrus*, *P. guajava*, and *Alnus* were least hazardous tree species. Also, 3 hazardous trees, 24 semihazardous trees, and 47 less hazardous trees were present in the area with hazard index 9, 6.04, and 2.91, respectively, necessitating the immediate attention of the relevant authorities. It was found that the existing management is inadequate and that hazardous trees need to be removed. It is further recommended to focus on tree management and species selection promoting research on location variables and their effects on species and their silvicultural characteristics, as well as their social and economic values. Also, ornamental species such as *Eucalyptus* and *Juniperus* and flowering species such as *Grevillea*, *Calliandra*, and *Jacaranda* are encouraged to be planted along the 2*∗*2 m spacing in an orderly manner. It is also essential for the Kathmandu district authorities to implement regular tree inspections, soil management practices, and defects and pest/diseases monitoring to safeguard public safety and maintain a healthy urban forest.

## Figures and Tables

**Table 1:** Hazard rating scores for categorization of trees and sites.

Hazard rating score		Category
For tree	For site	
1–4	1–4	Less hazardous
5–8	4–8	Semihazardous
9–12	8–12	Hazardous

**Table 2:** List of species recorded in the study area.

S. N.	Nepali name	English name	Scientific name	Family	Number
1	Kangiyo	Silk oak	*Grevillea robusta*	Proteaceae	18
2	Kapur	Camphor tree	*Cinnamomum camphora*	Lauraceae	12
3	Bar	Banyan	*Ficus bengaensis*	Moraceae	7
4	Others	—	*—*		6
5	Dhupi	Juniper	*Juniperus chinensis*	Cupressaceae	5
6	Peepal	Sacred fig	*Ficus religiosa*	Moraceae	5
7	Siris	Pink silk tree	*Albizzia julibrissin*	Fabaceae	4
8	Bakaino	Chinaberry tree	*Melia azedarach*	Meliaceae	2
9	Amala	Indian gooseberry	*Phyllanthus emblica*	Phyllanthaceae	1
10	Amba	Guava	*Psidium guajava*	Myrtaceae	1
11	Round bottlebrush	Red powder puff	*Calliandra hematocephala*	Fabaceae	1
12	Masala	Gum trees	*Eucalyptus camaldulensis*	Myrtaceae	1
13	Fir	Noble fir	*Abies procera*	Pinaceae	1
14	Jaiful	Winter jasmine	*Jasminum nudiflorum*	Oleaceae	1
15	Jamun	Black plum	*Syzygium cumini*	Myrtaceae	1
16	Kauli phool	Climbing hydrangea	*Pileostegia viburnoides*	Hydrangeaceae	1
17	Khari	Honeyberry	*Celtis australis*	Cannabaceae	1
18	Kimbu	Mulberry	*Morus alba*	Moraceae	1
19	Lahare Pipal	Cottonwood poplar	*Poplus deltoides*	Salicaceae	1
20	Nibuwa	Lemon	*Citrus limon Burm*	Rutaceae	1
21	Rudraksha	Stonefruit	*Elaeocarpus sphaericus*	Elaeocarpaceae	1
22	Syau	Apple	*Malus domestica*	Rosaceae	1
23	Uttis	Alder	*Alnus nepalensis*	Betulaceae	1

## Data Availability

The data used to support the findings of this study are included within the article.
